# Idiosyncrasy to Egg Albumin

**Published:** 1912-09

**Authors:** 


IDIOSYNCRASY TO EGG ALBUMIN.—Dr. Wm. P. Coues of Prouts Neck, Me. *Boston M. & S. Jour.*, Aug. 8, 1912, alludes to a similar report and bibliography by Schloss in the *Am. Jour. of Diseases of Children,* June, 1912. His own case was that of a child 21 months old, given egg white for the first time. Nausea and vomiting followed. A month later, an egg white was given on each of three successive days. After the last feeding, the child began to sneeze, had marked coryza and a general erythema with œdema of eye-lids and penis. No respiratory embarrassment, no fever, no marked prostration. Laxol internally and sodium bicarbonate externally gave relief promptly.

